# Flavonoid-Rich Extract of *Paulownia fortunei* Flowers Attenuates Diet-Induced Hyperlipidemia, Hepatic Steatosis and Insulin Resistance in Obesity Mice by AMPK Pathway

**DOI:** 10.3390/nu9090959

**Published:** 2017-08-30

**Authors:** Chanmin Liu, Jieqiong Ma, Jianmei Sun, Chao Cheng, Zhaojun Feng, Hong Jiang, Wei Yang

**Affiliations:** 1 School of Life Science, Jiangsu Normal University, No. 101, Shanghai Road, Tangshan New Area, Xuzhou 221116, Jiangsu, China; sunjianmei@jsnu.edu.cn (J.S.); chengchao@jsnu.edu.cn (C.C.); fzj@jsnu.edu.cn (Z.F.); jhxznu@126.com (H.J.); 15852113162@163.com (W.Y.); 2 School of Chemical Engineering, Sichuan University of Science and Engineering, No. 180, Huixing Road, Zigong 643000, Sichuan, China; jieqingma@126.com

**Keywords:** *Paulownia fortunei* flower, hyperlipidemia, hyperglycemia, obesity, hepatic fat accumulation, lipid metabolism, AMPK

## Abstract

The flavonoid-rich extract from *Paulownia fortunei* flowers (EPF) has been reported to prevent obesity and other lipid metabolism disease. However, the mechanism of its protective effects is not yet clear. The objective of this study was to investigate molecular factors involved in the hypoglycemic and hypolipidemic effects of EPF in obese mice fed a high-fat diet (HFD). Male h ICR (Institute of Cancer Research) mice were fed a HFD containing or not containing the EPF (50 or 100 mg/kg) for eight weeks. EPF reduced body weight gain, lipid accumulation in livers and levels of lipid, glucose and insulin in plasma as well as reduced insulin resistance as compared with the HFD group. EPF significantly decreased serum aminotransferase activity of the HFD group. We observed that EPF administration significantly increased the level of AMP-activated kinase (AMPK) phosphorylation and prevented fat deposits in livers and HepG2 cells, but these effects were blocked by compound C (an AMPK inhibitor). The protective effects of EPF were probably associated with the decrease in HMGCR, SREBP-1c and FAS expressions and the increase in CPT1 and phosphor-IRS-1 expressions. Our results suggest that EPF might be a potential natural candidate for the treatment and/or prevention of overweight and hepatic and metabolic-related alterations induced by HFD.

## 1. Introduction

High fat diet (HFD) could induce hepatic steatosis, hyperlipidemia, obesity, diabetes and other lipid metabolism disorder disease by regulating signal pathway of lipid metabolism in livers [1,2]. AMP-activated kinase (AMPK), a phylogenetically conserved serine/threonine protein kinase, is a key regulator of energy metabolic homeostasis and a crucial target for drugs both ancient and modern [3]. Activation of AMPK can play an important role in regulating energy balance and nutrient metabolism, such as the synthesis of fatty acids, cholesterol, glucose, and hepatic gluconeogenesis and translation. Moreover, AMPK is considered an attractive target for the prevention of diseases such as obesity, diabetes, inflammation and cancer [3,4]. It is reported that AMPK could be activated by natural phenolic compound, such as resveratrol, epigallocatechin gallate (EGCG), curcumin, quercetin, caffeic acid phenethyl ester (CAPE), berberine, and theaflavin [4].

Indigenous medicinal plants have been recommended for treatment of many metabolism diseases because of their easy availability and relatively fewer side effects [1,3,5]. Epidemiological studies revealed that an increased daily intake of phenolic compounds in dietary supplements act as anti-oxidant and anti-inflammatory agents to increase thermogenesis and energy expenditure while decreasing inflammation and oxidative stress, further supporting progress towards decreased metabolic disorders [1,5]. Previous studies confirmed that the species of the genus *Paulownia* (belonging to the family scrophulariaceae) exhibited a broad spectrum of biological effects, including antioxidant, anticarcinogenic [6,7], antiphlogistic [8], antiviral [9], antimicrobial [10] and anti-cholinesterase activities [11]. *Paulownia fortunei* (Seem.) Hemsl. is a fast growing ornamental tree, native to Mainland China and distributed almost all over the world [10]. This species is renowned as a polyphenol rich plant, which has been used in traditional Chinese medicine for the treatment of hypertension, enteritis, tonsillitis, bronchitis and dysentery [7,12,13]. The flowers of *P. fortunei* are also used as an additive to seasonal foods in China, made into a local delicacy called “Zheng Cai”. Previous studies have shown that flower extracts of *P. fortunei* (EPF) contain high amounts of flavonoids, which are mainly composed of apigenin, luteolin, hesperetin, β-sitosterol, thunberginol A, daucosterol, quercetin, kaempferol, and their derivatives [12,13]. In previous study, we showed that flavonoid puerarin could prevent hyperlipidemia, oxidative stress and other lipid metabolism disorder disease in livers [14,15]. For these reasons, we hypothesized that the extract from *P. fortunei* flowers (EPF) might play an important role in protecting HFD-induced hyperlipidemia, insulin resistance and hepatic fat accumulation. In this study, for the first time, we used high-fat diet induced obese mice to verify the protective effects of ethanol extract of *P. fortunei* flowers containing the flavonoids and to study the mechanisms focusing on these effects in enhancing AMPK signaling pathway and inhibiting lipid metabolism disorder in livers. 

## 2. Materials and Methods

### 2.1. Chemical Reagents

Antibodies against HMGCR (sc-27578), pMAPK (T172) (CST-2535), AMPK (CST-2532), pIRS-1 (Ser-307) (sc-101709), IRS-1 (sc-51517), FAS (sc-715), CPT1 (CST-12252), SREBP-1c (sc-13551) and β-actin (sc-1616) were obtained from Santa Cruz Biotechnology (Santa Cruz, CA, USA) or Cell Signaling Technology (Beverly, MA, USA). Rutin, luteolin, and apigenin (>99%) were obtained from Sigma Chemical Co. (Saint Louis, MO, USA). Quercetin 3-*O*-glucoside, luteolin 7-*O*-glucoside, kaempferol 3-*O*-glucoside, and apigenin 7-*O*-glucoside (>99%) were purchased from Shanghai Yongye Biotechnology Co., Ltd. or Shanghai Yuanye Biotechnology Co., Ltd. (Shanghai, China). All other solvents and reagents were purchased from Aladdin (Aladdin, Shanghai, China).

### 2.2. Sample Preparation

The flowers of *P. fortunei* were collected at Xuzhou (Jiangsu Province, China) in April 2016. Eight kilograms of dried flowers were pulverized into powders and extracted three times with 70% ethanol (5 L) at 40 °C for 48 h. After filtration and removal of residues, the extract was submitted to a spray-drying (180 °C inlet temperature and 100 °C outlet temperature). The isolated flavonoids were purified by Sephadex LH-20 column chromatography. Total flavonoid content was determined according to Samad et al. by the following colorimetric method, where catechin was used as a standard. Catechin concentrations ranging from 0.05 to 0.5 mg/mL were used to generate the standard calibration curve [16]. The product yield is 5.28%. The flower extracts of *P. fortunei* (EPF) contain high amounts of flavonoids (67.8% in EPF). 

The content (mg/g dry weight) of major flavonoids was determined by high performance liquid chromatography (HPLC) according to the method described with slight modifications [17,18] (Table 1). Briefly, HPLC analyses were performed on an Agilent 1100 series liquid chromatograph (Agilent Technologies, Santa Clara, CA, USA) consisting in a binary pump, an autosampler and a diode-array detector (DAD). A ZORBAX SB-C18 (4.6 × 150 mm, 5 μm particle diameter, Agilent Technologies, Santa Clara, CA, USA) column was employed. Gradient elution was performed with solution A, composed of 50 mM sodium phosphate (pH 3.3) and 10% methanol, and solution B, comprising 70% methanol, delivered at a flow rate of 1.0 mL/min as follows: initially 100% of solution A; for the next 15 min, 70% A; for another 30 min, 65% A; for another 20 min, 60% A; for another 5 min, 50% A; and finally 0% A for 25 min. The injection volume for the extract was 10 μL. A library was made, comprising retention times on HPLC and spectra of standard chemical compounds. The extracts were then analyzed using the same HPLC system. The detected polyphenol peaks were compared with respect to retention time with those in the library.

### 2.3. Cell Culture and Treatments

Human HepG2 hepatocytes were cultured and treated previously described [19]. Briefly, human hepatoma-derived HepG2 cells were maintained in low glucose-containing Dulbecco’s modied Eagle’s medium. After reaching 75% confluence, the cells were serum-starved for 16 h and then exposed to FFA (free fatty acids) to induce fat overloading. The cells were treated with EPF (10–100 g/mL) or FFA (0.25–2 mM). Cellular triglyceride (TG) was detected with related kits as manufacturer’s instructions (Nanjing Jiancheng Bioengineering Institute, Nanjing, China).

### 2.4. Animals and Experimental Procedure

The present research reported in this paper was conducted in accordance with the Chinese legislation and NIH publications on the use and care of laboratory animals. Relevant university committees for animal experiments approved these experiments, ethic approval number: GB14925-2001; JSNUCAE-2017-12.

Male ICR mice (20–25 g) were purchased from the Branch of National Breeder Center of Rodents (Beijing, China). Mice were maintained in an environmentally controlled room (23 ± 2 °C, 55 ± 10% humidity) for 1 week for acclimatization. Then, fifty mice were randomly assigned to five groups (10 mice/group).

Group I (vehicle control) mice were fed a standard normal chow diet (SND) consisting of 60% kcal carbohydrate, 24% kcal protein, and 16% kcal fat, with a total energy of 3.1 kcal/g. Mice in Group II, Group III and Group IV were fed a high fat diet (HFD) consisting of 21.3% kcal carbohydrate, 18.4% kcal protein, and 60.3% kcal fat, with a total energy of 5.1 kcal/g. Mice in Group III and Group IV were fed a high fat diet (HFD) and daily given EPF in distilled water containing 0.1% Tween 80 at two doses 50 and 100 mg/(kg day), respectively. Mice in Group V (EPF 100 mg/kg) were fed a standard normal chow diet (SND) and received EPF in distilled water containing 0.1% Tween 80 at a dose of 100 mg/(kg day) by oral gavage. The EPF concentration was set to the maximum concentration that had not affected the food intake in a preliminary experiment [20]. The food intake and body weight of the animals were measured daily.

The experiment lasted for eight weeks. At the end of treatment, mice were sacrificed and blood samples were drawn by cardiac puncture with heparined tubes. The plasma was separated by centrifugation (3000× *g* for 10 min at 4 °C) and stored at −70 °C until analysis. The liver tissues was immediately excised for experiments or stored at −70 °C for later use. 

### 2.5. Biochemical Analysis

The levels of serum glucose, total cholesterol (TC), triglyceride (TG), density lipoprotein (LDL), high-density lipoprotein (HDL), urea and creatinine and the activities of alanine aminotransferase (ALT) and aspartate aminotransferase (AST) were determined using a commercial kit (Nanjing Jiancheng Bioengineering Institute, Nanjing, China) [14,15]. 

### 2.6. Oral Glucose Tolerance

Oral glucose tolerance was determined as described previously [21]. Insulin levels were characterized by a corresponding mouse ELISA kit according to the manufacturer’s instructions. Insulin resistance (IR) and the homeostatic index of insulin resistance (HOMA-IR) were determined as described previously (KingMed Diagnostics, Gongzhou China) [21].

### 2.7. Oil Red O Staining

Oil Red O was used to stain intracellular lipids as described previously [19]. Briefly, HepG2 cells in different groups were cultured in the corresponding medium for 24 h. Cells were then fixed with 4% paraformaldehyde and stained with a freshly prepared working solution of oil red O at room temperature. The histological changes of liver were evaluated using a commercial kit, stained by the Oil Red O solution (0.5%; Nanjing Jiangcheng Bioengineering Institute, Nanjing, China) according to the manufacturer’s protocol. 

### 2.8. Measurement of Liver Triglyceride Content

Liver TG content was determined as described previously [14,22]. The levels of hepatic TG in the extraction solution were determined by enzymatic methods using commercially available kits (Elabscience Biotechnology, Wuhan, Hubei, China) according to the instructions of the manufacturer.

### 2.9. Western Blot Analyses

To measure the effect of EPF on gene expression in mouse livers, Western blot analysis was performed as previously described by us [14,15]. Total protein content was determined by BCA protein assay (Thermo Scientific Pierce, Rockford, IL, USA).

### 2.10. Statistical Analysis

Results were expressed as mean ± standard error (SE). Significant differences among the groups were assessed by one-way ANOVA with Tukey’s post hoc test (ANOVA; *p* < 0.05).

## 3. Results

### 3.1. Identification of Purified EPF

We find that flower extracts of *P. fortunei* (EPF) contain high amounts of flavonoids (67.8% in EPF). As shown in Table 1, using standard chemical compound of flavonoids, seven kinds of flavonoids were identified in the extract from *P. fortunei* flowers. Apigenin and luteolin 7-*O*-glucoside were major flavonoids in the extract of *P. fortunei* flowers.

### 3.2. General Characteristics

Table 2 shows that mice fed HFD gained more body weight as compared with the control group (*p* < 0.05). The average daily food intake per mouse was greater in Group I (control) than that in Group II (HFD group). Since the calorie density of standard normal chow diet is less than that of high fat diet, the average daily calorie intake per mouse was markedly less in the control group as compared with the HFD group (*p* < 0.05). However, treatment with EPF 50 and 100 mg/kg significantly ameliorated HFD-induced additional weight gain and calorie intake. The standard normal chow diet supplemented with EPF caused a slight decrease in body weight compared to the standard normal chow diet only, but this decline did not show a significant difference.

### 3.3. Liver Damage Parameters

To determine whether EPF can attenuate the liver damage in the HFD mice, we measured the aminotransferase activities. As shown in Table 3, Fat-rich diet led to higher serum aminotransferase activities of ALT (83.3%), AST (42.3%) as compared with the control group, but these effects were blocked by EPF supplementation. No significant differences in the aminotransferase activities in serum were found between the normal diet supplied with the EPF group and the control group.

### 3.4. Plasma Glucose and Insulin Concentrations

As shown in Table 3, a HFD induced marked elevations of blood glucose (101.1%), plasma insulin (61.2%) and HOMA-IR (169.8%), while EPF treatment markedly lowered fasting plasma insulin, glucose and HOMA-IR relative to HFD mice. No significant differences in blood glucose, plasma insulin and HOMA-IR were found between the normal diet supplied with the EPF group and the control group.

### 3.5. Serum Lipid Profiles

As expected, mice fed with HFD exhibited a significant hyperlipidemia characterized by increased TC (141.8%), TG (89.2%) and LDL (541.5%) levels in serum compared with these of the control group, respectively (Table 3). Notably, EPF treatment markedly decreased serum TC, TG and LDL levels in HFD mice after eight weeks (*p* < 0.01). Moreover, HDL level was significantly increased (*p* < 0.05) in the group co-treated with HFD and EPF compared with the animals only treated with HFD (*p* < 0.05). No significant differences in blood lipid levels were found between the normal diet supplied with the EPF group and the control group.

### 3.6. Hepatic Lipid Accumulation

**Figure 1:**
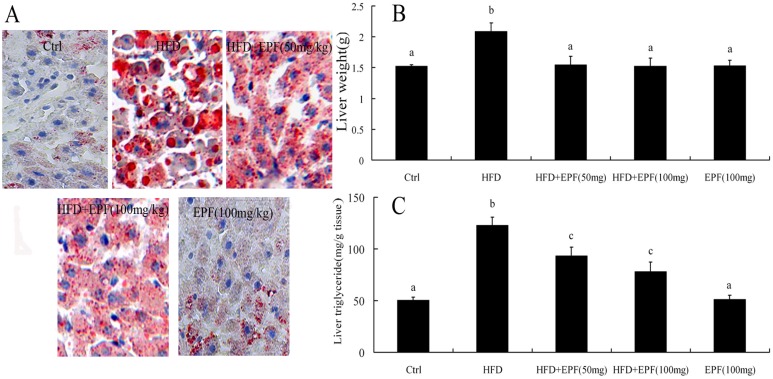
Hepatic lipid accumulation, liver weight and histological evaluation in mice: (**A**) the status of hepatic lipid accumulation in the different rat groups was analyzed after liver section staining with the Oil Red O method; (**B**) liver weight; and (**C**) liver triglyceride. SND, standard normal chow diet group (Control, low chow diet group); HFD, high fat diet group; HFD + EPF, high fat diet and the extracts from *P. fortunei* flowers (50 or 100 mg/kg). Original magnification, ×200. Significant differences among the groups were assessed by one-way ANOVA with Tukey’s post hoc test Values are mean ± the standard error of the mean (SEM) (*n* = 3). Values that do not share a common superscript (a,b,c) differ significantly at *p* ≤ 0.05.

In HFD group, the levels of liver weight and hepatic TG were increased by 37.4% and 144.8% as compared with those of the control group, respectively (*p* < 0.01). However, EPF treatment reduced hepatic lipid accumulation and liver weight (Figure 1B,C). No significant differences in liver TG content were found between the normal diet supplied with the EPF group and the control group. Tissue sections stained with oil red O represented that a signification increase in the hepatic levels of lipid deposition in HFD-fed mice compared with those of the control group. Likewise, EPF treatment attenuated hepatic lipid accumulation, consistent with liver TG content (Figure 1A).

### 3.7. Hepatic AMPK Activation

**Figure 2:**
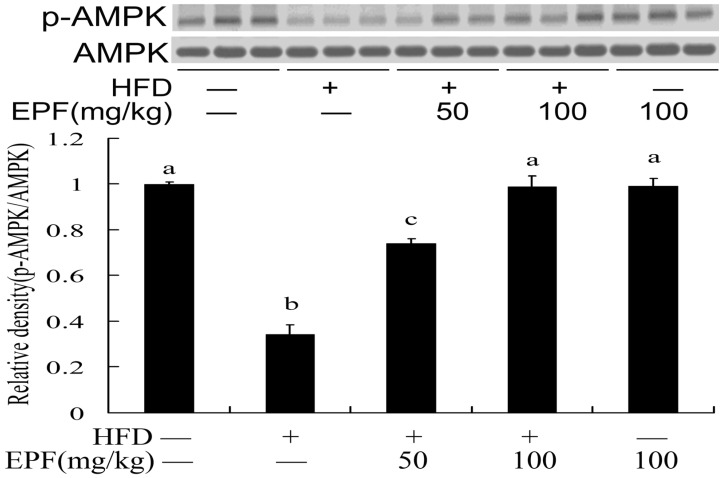
EPF increased AMPK activations in livers of HFD-fed. The vehicle control is set as 1.0. Significant differences among the groups were assessed by one-way ANOVA with Tukey’s post hoc test. Each value is expressed as mean ± SEM (*n* = 3). Values that do not share a common superscript (a,b,c,d) differ significantly at *p* ≤ 0.05.

To reveal the potential mechanisms of EPF action, we examined the AMPK phosphorylation levels in livers of HFD-fed mice. Figure 2 showed that the AMPK phosphorylation levels significantly decreased in livers of HFD-fed mice. However, the levels of phosphorylated AMPK were reduced by treatment with EPF (50 or 100 mg/kg).

### 3.8. AMPK Activation in HepG2 Cells

**Figure 3:**
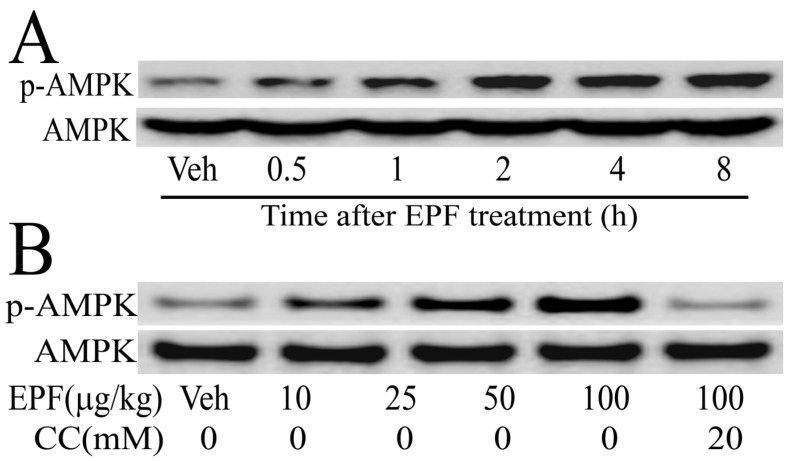
Western blot analysis of protein expression levels of AMPK in HepG2 cells: (**A**) cellular extracts were collected at the indicated times after treatment of EPF (100 μg/mL); and (**B**) cellular extracts were collected at 4 h after EPF treatment (10–100 μg/mL).

We further examined the AMPK phosphorylation levels in HepG2 cells. As shown in Figure 3, the AMPK phosphorylation levels continuously increased until 8 h in HepG2 cells, when treated with 100 μg/mL EPF (Figure 3A). Moreover, HepG2 cells were treated with 10–100 μg/mL EPF for 4 h. expression levels of phosphorylated AMPK increased in a dose-dependent manner. However, after treatment with compound C (an AMPK inhibitor), the phosphorylated AMPK levels in HepG2 cells were significantly reduced (Figure 3B). These results clearly show that EPF treatment leads to AMPK phosphorylation.

### 3.9. AMPK Inhibition Reduces the Effect of EPF on Lipid Accumulation in FFA-Exposed Hepatocytes

**Figure 4:**
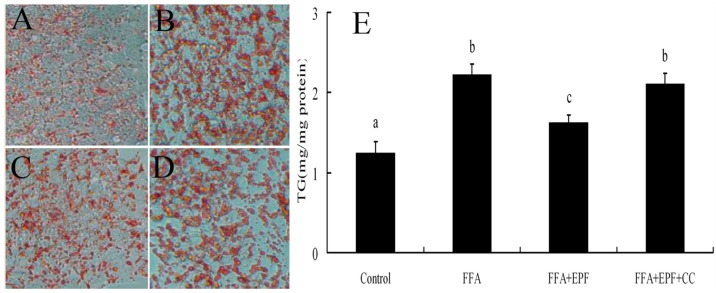
AMPK inhibition reduces the effect of EPF on lipid accumulation in FFA (free fatty acids) exposed hepatocytes: (**A**) control cells; (**B**) FFA treated cells; (**C**) FFA + EPF treated cells; (**D**) FFA + EPF + Compound C treated cells; amd (**E**) the level of intracellular TG in HepG2 cells. The cells were treated with 1 mM FFA mixture for 24 h and EPF (100 μg/mL) was treated 1 h prior to FFA mixture exposure. The cells were stained with Oil Red O and analyzed using a spectrometer at 545 nm. DMSO (0.1%) was treated as a vehicle for EPF, and control cells were treated only with 1% BSA. Compound C (CC) was pretreated 30 min prior to EPF treatment. Photographs (Original magnification, ×400) are representative images of 3 independent experiments. Values that do not share a common superscript (a,b,c) differ significantly at *p* ≤ 0.05 (DMRT).

We then examined the effects of AMPK activation on lipid accumulation in FFA-exposed hepatocytes. HepG2 cells were treated with compound C 30 min prior to EPF treatment. Lipid accumulation in HepG2 cells was measured 24 h after FFA exposure using Oil Red O staining. EPF markedly inhibited lipid accumulation in FFA-exposed HepG2 cells at a concentration of 100 μg/mL. Moreover, FFA exposure markedly increased intracellular TG levels in HepG2 cells by 78.8% as compared with these of the control group, but these effects were blocked by compound C (Figure 4).

### 3.10. Hepatic Expressions of Proteins Associated with Lipid Metabolism in Livers

**Figure 5:**
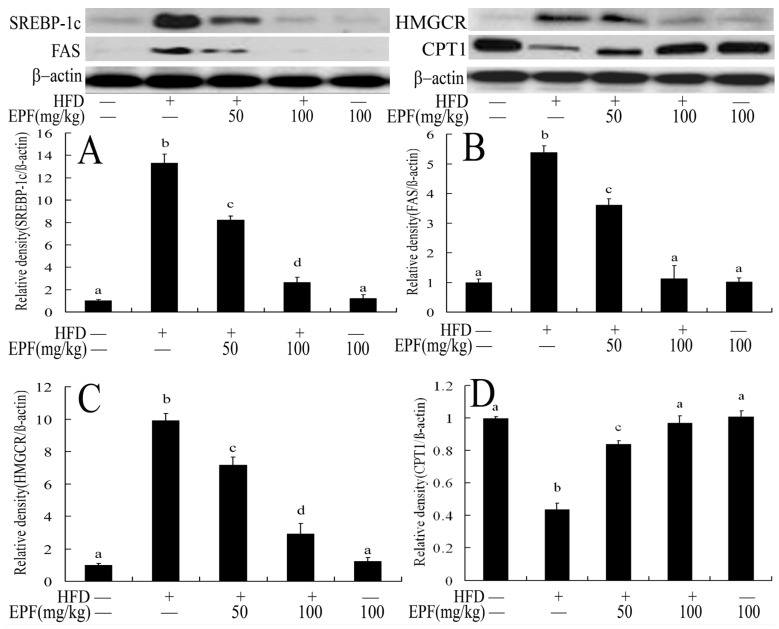
Western blot analysis of protein expression levels in association with the hepatic lipid metabolism in mice: (**A**) relative density analysis of HREBP-1c; (**B**) relative density analysis of FAS; (**C**) relative density analysis of HMGCR; and (**D**) relative density analysis of CPT1. β-Actin was probed as an internal control in relative density analysis. The vehicle control is set as 1.0. Significant differences among the groups were assessed by one-way ANOVA with Tukey’s post hoc test. Each value is expressed as mean ± SEM (*n* = 7). Values that do not share a common superscript (a,b,c,d) differ significantly at *p* ≤ 0.05.

To investigate the mechanisms through which EPF regulated hepatic lipid accumulation, we further evaluated the expressions of proteins associated with lipid metabolism in livers. As shown in Figure 5, the expression levels of sterol regulatory element binding protein 1c (SREBP-1c), 3-hydroxy-3-methylglutaryl-CoA reductase (HMGCR) and fatty acid synthase (FAS) were markedly up-regulated in the livers of HFD group as compared with the control group. However, EPF supplementation down-regulated the expression levels of SREBP-1c protein, and its target genes (*p* < 0.01). The carnitine palmitoyltransferase 1 (CPT1) expression level was also decreased in the livers of HFD group as compared with the control group. Interestingly, EPF supplementation significantly enhanced the CPT1 expression in the livers of HFD group (*p* < 0.01). 

### 3.11. Hepatic Activation of IRS-1

**Figure 6:**
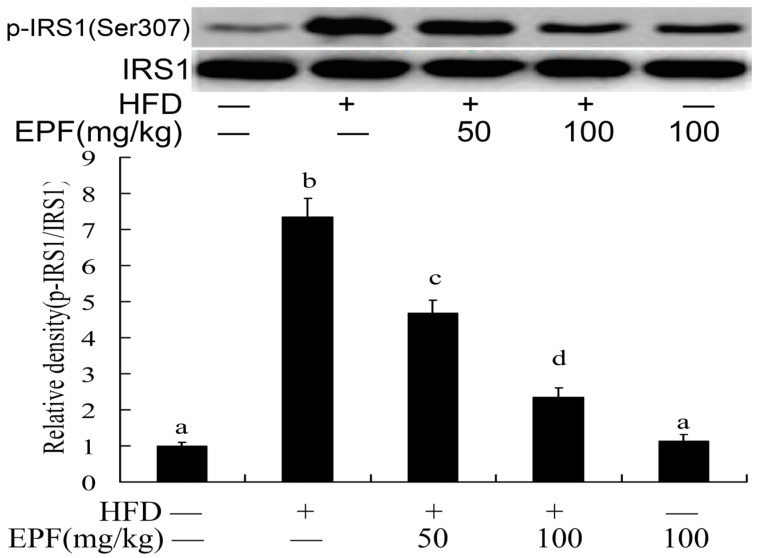
Western blot analysis of IRS1 phosphorylation levels in the livers of mice. The relative density is expressed as the ratio (Phospho-IRS1/IRS1). The vehicle control is set as 1.0. Significant differences among the groups were assessed by one-way ANOVA with Tukey’s post hoc test. Each value is expressed as mean ± SEM (*n* = 7). Values that do not share a common superscript (a,b,c) differ significantly at *p* ≤ 0.05.

The activation of insulin receptor substrates 1 (IRS1) played important role in blood glucose regulation in livers. To investigate the mechanisms through which EPF inhibited insulin resistance in HFD group, we evaluated the IRS-1(Ser 307) phosphorylation level in mouse livers. As shown in Figure 6, the IRS-1(Ser 307) phosphorylation levels were markedly increased in the livers of HFD group as compared with the control group. Interestingly, EPF supplementation significantly enhanced the IRS-1(Tyr 307) phosphorylation in the livers of HFD-fed mice (*p* < 0.01).

## 4. Discussion

The flower of *P. fortunei* has long been used a medicine and food source in China. However, detailed information related to the beneficial effects of its polyphenol composition remains scarce. Here, we aimed to evaluate the protective effects of extract from *P. fortunei* flowers (EPF) on insulin resistance and lipid metabolism disorders in livers. We found that flower extracts of *P. fortunei* (EPF) contain high amounts of flavonoids. The current study clearly indicated that EPF inhibited hyperlipidemia, hepatic lipid accumulation and insulin resistance in obese mice by AMPK pathway.

Several studies had revealed that high fat diet could cause hepatic steatosis, hyperlipidemia, hyperinsulinemia, obesity, insulin resistance and other lipid metabolism disorder disease in humans and in laboratory animals [1,2]. The results of the present study showed that high-fat diet led to significant increase in body weight, hyperlipidemia, hyperglycemia, hyperinsulinemia, and hepatic lipid accumulation, which were in agreement with previous studies [1,2,23]. Phenolic compounds, which are widespread in plants, showed therapeutic effects to obesity and other HFD-induced disease [1,24]. In this study, as shown in Table 2, we observed that supplementation with EPF (50 and 100 mg/kg) significantly deceased body weight by 23.42% and 31.26% compared with HFD mice, respectively, which suggested that EPF has the anti-obesity effects. 

High fat diet could induce liver damage [2,25,26]. Previous research found that the flower extracts of the Scrophulariaceae family plants had protective effects against hepatotoxicity [26]. Consistently, we found that EPF supplementation significantly decreased the diagnostic indicators of liver damage as compared with the fat-rich diet (Table 3). These results suggest susceptibility of EPF to the harmful action of the fat-rich diet, and the protective effect of EPF. 

It is well known that long-term HFD could induce hyperglycemia and elevations of blood insulin levels. Moreover, increased insulin secretion is in part related to pancreatic islet hyperplasia with progression of insulin-resistance by HFD supply [5,22]. In the present study, we found that blood glucose, insulin levels and HOMA-IR increased significantly (*p* ≤ 0.05) in the HFD group compared to the control group, revealing insulin resistance in the HFD group (Table 3). Furthermore, this rising blood glucose levels may be attributed to the large accumulation of fat in the liver and the enhancement of gluconeogenesis induced by hepatic cells, reducing the transformation of glucose into fat caused by lipodystrophy [27,28]. However, EPF supplementation in HFD-induced obesity mice for eight weeks markedly ameliorated insulin resistance conditions, as indicated by lower serum glucose and insulin levels at the end of the experiment compared to the HFD group, which is thought to stimulate the pancreatic insulin secretion from the β cells of the islets of langerhans and aldose reductase enzyme inhibitory activity [20]. The rich flavonoids (apigenin, luteolin, rutin and hesperetin) in EPF might play important role in the anti-obesity and hypoglycemic effects [24,29,30]. The decreased insulin levels in mice supplied with EPF may be attributed to reduced body weight gain and fat accumulation in livers. Moreover, the protective effects of EPF against obesity and lipid accumulation could also be attributed to the prevention of hyperlipidemia, hyperglycemia in mice fed with HFD. Thus, EPF could inhibit insulin-resistance or conduce to the classical diabetes mellitus treatment.

Previous research from our laboratory and others illustrated that several flavonoids including puerarin, quercetin, hesperetin, epicatechin, apigenin and anthocyanins could reduced serum lipid levels [14,15,24,31]. Previous reports have revealed different results regarding the change in serum TG, TC and LDL-C levels of ICR mice treated with HFD for eight weeks [32,33,34]. Present observations are in agreement with previous demonstrations that HFD increased the levels of TG, TC and LDL-C [33,34]. Interestingly, EPF treatment markedly decreased serum TC, TG and LDL levels and increased HDL levels in HFD mice after eight weeks, which suggested that flavonoids might be the main bioactive compounds in EPF that exhibited the beneficial effects on the lipid profile (Table 3). Furthermore, the digestion and intestinal absorption of the flavonoids apigenin, luteolin, quercetin and hesperetin from EPF might lower hepatic lipid accumulation suppressing hepatic lipogenesis and lipid absorption [29,30,31]. We observed that EPF treatment significantly decreased liver weight and liver triglyceride content of mice in a dose-dependent manner. Histological analysis showed that EPF supplementation markedly reduced lipid vacuoles and lipid droplets in livers of HFD-fed mice (Figure 1), consistent with liver TG content. 

AMPK plays important roles in regulating energy status and lipid metabolism, which is also potential therapeutic target for many prevalent diseases, including diabetes, obesity, and high blood pressure [3,4]. Many studies showed that several natural compounds, including resveratrol, epigallocatechin gallate, berberine, and quercetin could inhibit lipid-related metabolic disorders by regulating the AMPK activition and its target genes [3,4,19]. In this study, EPF markedly stimulated the AMPK activation in livers of mice and in HepG2 cells (Figure 2 and Figure 3). This result suggested that AMPK pathway may be involved in the modulation of hepatic lipid metabolism in livers of HFD-fed mice and HepG2 cells treated with EPF. Consistent with this regulation, the AMPK inhibitor compound C blocked the effects of EPF on lipid accumulation in HepG2 cells, demonstrating that AMPK activation is necessary for the modulation of hepatic lipid metabolism (Figure 4). 

AMPK can mediate lipid metabolism in livers by regulating the expression levels its target genes SREBP-1c, FAS, ACC and HMGCR [3,4,19]. Previous report showed that AMPK coordinates the long-term adaptation of lipid metabolism by regulating the transcriptional factor SREBP-1c (a key transcription factor involved in the control of cholesterol and fatty acid synthesis), which further increase the transcription of FAS and SCD-1, resulting in an increase in the synthesis of TG [3,4,19,35]. Several studies have demonstrated that the flavonoid extracted from plants could inhibit lipid-related metabolic disorders by regulating the expression levels of AMPK and SREBP-1c and their target genes [1,22,29,36]. As demonstrated in our Western blot analysis, EPF supplementation significantly down-regulated the expression levels of AMPK and SREBP-1c and their target gene HMGCR, an endoplasmic reticulum bound and peroxisomal enzyme that is the rate-limiting step in cholesterol biosynthesis, suggesting that EPF had a positive effect due to the lower expression of HMGCR and contributed to inhibit the serum cholesterol increase due to high fat diet (Figure 5). Moreover, EPF treatment significantly down-regulated the expression level of FAS, a key enzymes involved in lipogenesis and up-regulated the expression level of carnitine palmitoil transferase 1 (CPT1), which catalyzes the entrance of fatty acids into the mitochondria and it is the rate limiting enzyme of hepatic fatty acid β-oxidation (Figure 5). Therefore, these results implied that the reduced biosynthesis and enhanced fatty acid oxidation might contribute to the beneficial effects of EPF on hyperlipidemia and hepatic lipid accumulation in the HFD mice. These data explained the anti-obesity, hypolipidemic and hypoglycemic effects of EPF to a certain extent.

As previously expressed, insulin resistance is another key pathophysiological feature and pathogenesis in high fat diet mice [2,20,21]. The hyperlipidemia and hyperglycemia induced by high fat diet could decrease the number of insulin receptors, glucose transport and metabolism thereby increasing insulin resistance and reducing insulin sensitivity [23]. Insulin signaling is a cascade of events initiated by the activation of insulin receptor substrates (IRS). AMPK could regulate glucose metabolism insulin resistance by IRS pathway [37]. Insulin resistance could also decrease the AMPK phosphorylation and increase the expression levels of SREBP-1c, which further suppressed IRS-1 activation, inhibits IRS-1-associated insulin signaling and thereby decreases glucose uptake and utilization [3,4,19,38]. To understand the molecular mechanisms contributing to insulin resistance, we further observed the effects of EPF supplementation on these key factors of insulin signaling in liver tissue (Figure 6). Consistent with these reports, our results showed HFD feeding for 8 weeks increased the SREBP-1c expression levels and inhibited its downstream molecule IRS-1 (Ser 307) phosphorylation, which substantiates the development of insulin resistance in the livers of mice. However, EPF dietary supplementation partially or completely prevented all these alterations associated with HFD consumption, suggesting that EPF might be a natural flavonoid-rich extract and the activator of IRS-1, which in turn regulates the expression of downstream genes involved in hepatic glucose and lipid metabolism to lower blood glucose and lipid.

## 5. Conclusions

**Figure 7:**
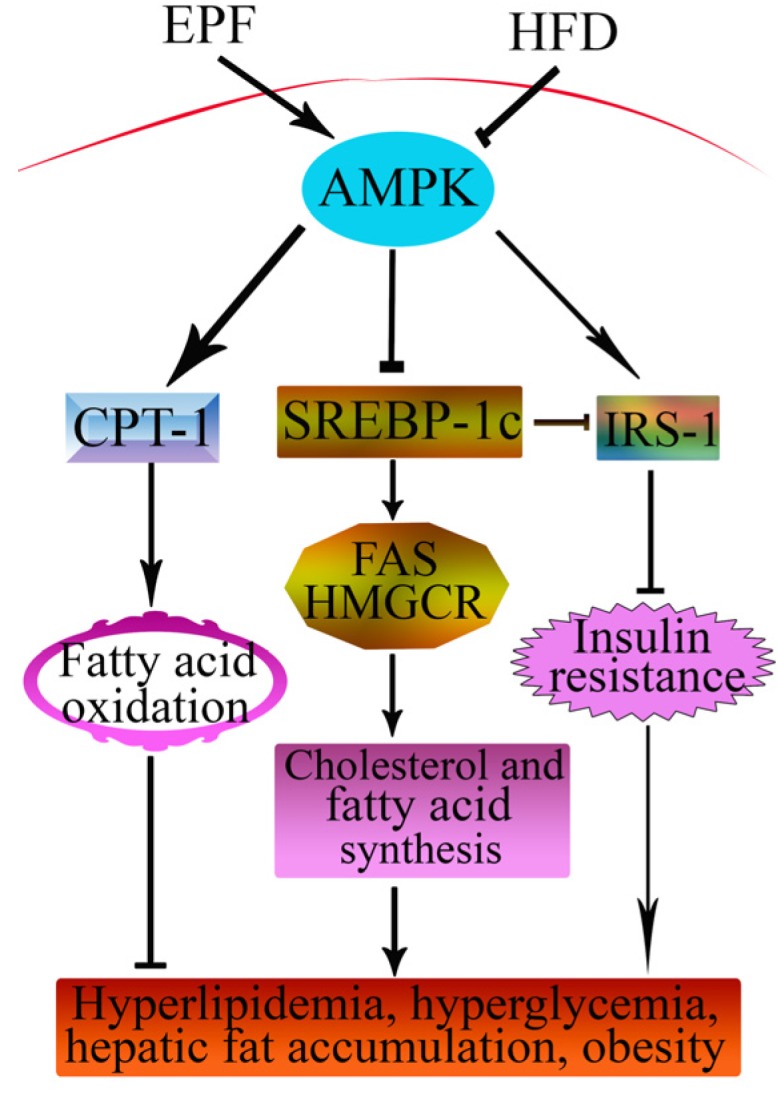
Schematic diagram showing protective signaling of EPF in livers of HFD-fed mice. The → indicates activation or induction, and ┤indicates inhibition or blockade

This is the first report that the extracts from *P. fortunei* flowers have potent protective effects against hyperlipidemia, hepatic lipid accumulation and insulin resistance in HFD mice and the protective effects of EPF, at least in part, were associated with the decreased lipogenesis, increased glucose metabolism and induced fatty acid oxidation in livers by AMPK pathway. We propose a possible protective effect of EPF (Figure 7). Our results emphasize the importance of dietary intervention in the treatment and/or prevention of diseases induced by HFD. Although, seven flavonoid compounds were identified in the extract from *P. fortunei* flowers by HPLC analysis, we were not clear if all of them were bioactive agents. Therefore, this question warrants further investigation.

## Figures and Tables

**Table 1:** Content of major flavonoid compounds in the flower extracts of *P. fortunei* (EPF) used in the study.

Compound	Content (mg/g Dry Weight)
Apigenin	16.63 ± 0.01
Luteolin	7.16 ± 0.04
Rutin	1.87 ± 0.02
Luteolin 7-*O*-glucoside	9.26 ± 0.02
Kaempferol 3-*O*-glucoside	5.17 ± 0.01
Apigenin 7-*O*-glucoside	4.13 ± 0.03
Quercetin 3-*O*-glucoside	3.95 ± 0.04

Data are expressed as mean ± standard error (SE) (*n* = 3). Significant differences among the groups were assessed by one-way ANOVA with Tukey’s post hoc test.

**Table 2:** Effect of EPF on the body weight gain, food intake and calorie intake.

Parameter	SND	HFD	HFD + EPF (50 mg/kg)	HFD + EPF (100 mg/kg)	SND + EPF (100 mg/kg)
Body weight gain (g)	10.26 ± 1.93 ^a^	17.85 ± 3.12 ^b^	13.67 ± 3.04 ^c^	12.27 ± 2.71 ^c^	10.09 ± 2.18 ^a^
Food intake (g/day)	4.82 ± 0.15 ^a^	3.93 ± 0.22 ^b^	3.41 ± 0.13 ^c^	3.42 ± 0.29 ^c^	4.79 ± 0.24 ^a^
Calorie intake (kcal/g/day)	14.94 ± 0.47 ^a^	20.04 ± 1.12 ^b^	17.39 ± 0.66 ^c^	17.44 ± 1.48 ^c^	14.85 ± 0.75 ^a^

Significant differences among the groups were assessed by one-way ANOVA with Tukey’s post hoc test. Data represent mean ± standard error (SE) of 10 individual mice; values that do not share a common superscript (a,b,c) differ significantly at *p* ≤ 0.05. SND, standard normal chow diet group (Control, low chow diet group); HFD, high fat diet group; HFD + EPF, high fat diet and the extracts from *P. fortunei* flowers (50 or 100 mg/kg).

**Table 3:** Effect of EPF on serum biochemical parameters of mice.

Parameter	SND	HFD	HFD + EPF (50 mg/kg)	HFD + EPF (100 mg/kg)	SND + EPF (100 mg/kg)
ALT (U/L)	28.32 ± 1.38 ^a^	51.91 ± 2.16 ^b^	42.36 ± 1.83 ^c^	36.51 ± 2.11 ^d^	28.43 ± 2.07 ^a^
AST (U/L)	42.86 ± 2.14 ^a^	60.97 ± 1.83 ^b^	44.73 ± 3.21 ^c^	43.68 ± 2.54 ^c^	42.89 ± 3.19 ^a^
Glucose (mM)	7.65 ± 0.48 ^a^	15.38 ± 2.05 ^b^	11.12 ± 1.25 ^c^	9.93 ± 1.01 ^d^	7.68 ± 0.27 ^a^
Insulin (mU/L)	3.38 ± 0.17 ^a^	5.45 ± 1.32 ^b^	4.26 ± 0.42 ^c^	3.82 ± 0.24 ^d^	3.39 ± 0.23 ^a^
HOMA-IR	1.16 ± 0.02 ^a^	3.73 ± 0.04 ^b^	2.11 ± 0.05 ^c^	1.69 ± 0.03 ^d^	1.16 ± 0.01 ^a^
TC (mM)	2.92 ± 0.21 ^a^	7.06 ± 0.34 ^b^	5.27 ± 0.19 ^c^	4.46 ± 0.32 ^d^	2.91 ± 0.16 ^a^
TG (mM)	0.83 ± 0.11 ^a^	1.57 ± 0.14 ^b^	1.19 ± 0.12 ^c^	0.97 ± 0.09 ^d^	0.82 ± 0.14 ^a^
HDL (mM)	1.59 ± 0.15 ^a^	1.36 ± 0.13 ^b^	1.49 ± 0.11 ^c^	1.53 ± 0.12 ^a^	1.59 ± 0.21 ^a^
LDL (mM)	0.41 ± 0.06 ^a^	2.63 ± 0.21 ^b^	1.67 ± 0.13 ^c^	1.54 ± 0.17 ^c^	0.40 ± 0.09 ^a^

Significant differences among the groups were assessed by one-way ANOVA with Tukey’s post hoc test. Data represent mean ± standard error (SE) of seven individual mice; values that do not share a common superscript (a,b,c,d) differ significantly at *p* ≤ 0.05. SND, standard normal chow diet group (Control, low chow diet group); HFD, high fat diet group; HFD + EPF, high fat diet and the extracts from *P. fortunei* flowers (50 or 100 mg/kg).

## Abbreviations

ALT	alanine aminotransferase
AST	aspartate aminotransferase
AMPK	AMP-activated kinase
CPT1	the carnitine palmitoyltransferase 1
EPF	the extract from *Paulownia fortunei* flowers
FAS	fatty acid synthase
HFD	high fat diet
HMGCR	3-hydroxy-3-methylglutaryl-CoA reductase
IRS-1	insulin receptor substrate 1
SREBP-1c	sterol regulatory element binding protein 1c
